# Traditional Medicinal Uses, Phytochemistry, Biological Properties, and Health Applications of *Vitex* sp.

**DOI:** 10.3390/plants11151944

**Published:** 2022-07-26

**Authors:** Nurkhalida Kamal, Nurul Syahidah Mio Asni, Ivana Nur Allisya Rozlan, Muhammad Aniq Hamzah Mohd Azmi, Noor Wini Mazlan, Ahmed Mediani, Syarul Nataqain Baharum, Jalifah Latip, Suvik Assaw, Ru Angelie Edrada-Ebel

**Affiliations:** 1 Institute of Systems Biology (INBIOSIS), Universiti Kebangsaan Malaysia (UKM), Bangi 43600, Malaysia; nurul.syahidah168@gmail.com (N.S.M.A.); ivananurallisyarozlan@gmail.com (I.N.A.R.); medianiahmed47@gmail.com (A.M.); nataqain@ukm.edu.my (S.N.B.); 2 Analytical and Environmental Chemistry Unit, Faculty of Science and Marine Environment, Universiti Malaysia Terengganu, Kuala Nerus 21030, Malaysia; aniqhamzah12@gmail.com; 3 Institute of Marine Biotechnology, Universiti Malaysia Terengganu, Kuala Nerus 21030, Malaysia; aasuvik@umt.edu.my; 4 Department of Chemistry, Faculty of Science and Technology, Universiti Kebangsaan Malaysia (UKM), Bangi 43600, Malaysia; jalifah@ukm.edu.my; 5 Marine Biology Unit, Faculty of Science and Marine Environment, Universiti Malaysia Terengganu, Kuala Nerus 21030, Malaysia; 6 Strathclyde Institute of Pharmacy and Biomedical Sciences, University of Strathclyde (SIPBS), The John Arbuthnott Building, 161 Cathedral Street, Glasgow G4 0RE, UK; ruangelie.edrada-ebel@strath.ac.uk

**Keywords:** *Vitex*, tradional medicinal use, phytochemistry, biological activity, health application

## Abstract

The genus *Vitex* is also known as a chaste tree, in which it is a large shrub native to the tropical and subtropical regions of the world. A diverse range of species is distributed throughout Southern Europe, the Mediterranean, and Central Asia. The *Vitex* tree, including its leaves and fruits, has been used for herbal remedies in the form of pastes, decoctions, and dried fruits since ancient times. This article aimed to prepare a comprehensive review of traditional uses and secondary metabolites derived from *Vitex* sp., including the chemical compounds, biological activities, application of *Vitex* in human clinical trials, toxicology and safety, marketed products, and patents. The scientific findings were obtained using a number of search engines and databases, including Google Scholar, PMC, and ScienceDirect. *Vitex* species are well known in pharmacology to have medicinal values, such as anti-inflammatory, antibacterial, antifungal, antimicrobial, antioxidant, and anticancer properties. Previous studies reported that some species are proven to be effective in treating diseases, such as diabetes, and improving female health. A total of 161 compounds from different *Vitex* species are reported, covering the literature from 1982 to 2022. A chemical analysis report of various studies identified that *Vitex* exhibited a wide range of phytoconstituents, such as iridoid, diterpenoid, ecdysteroid, and flavonoid and phenolic compounds. Apart from that, the review will also discuss the application of *Vitex* in human clinical trials, toxicology and safety, marketed products, and patents of the genus. While the extracts of the genus have been made into many commercial products, including supplements and essential oils, most of them are made to be used by women to improve menstrual conditions and relieve premenstrual syndrome. Among the species, *Vitex agnus-castus* L. is the only one that has been reported to undergo clinical trials, mainly related to the use of the genus for the treatment of mastalgia, menstrual bleeding problems, amenorrhea, menorrhagia, luteal insufficiency, and premenstrual syndrome. Overall, the review addresses recent therapeutic breakthroughs and identifies research gaps that should be explored for prospective research work.

## 1. Introduction

*Vitex* is one of the largest genera in the Lamiaceae family (formerly under the family Verbenaceae), consisting of 217 species in total [1]. Some of the most well recognized and researched species of *Vitex* are *Vitex negundo* L., *Vitex agnus-castus* L., *Vitex trifolia* L, *Vitex rotundifolia*, *Vitex cymosa* Bertero ex Spreng, and *Vitex peduncularis* Wall. Ex Schauer. They are scattered all over the world and can be primarily found in tropical regions, except for a few instances in subtropical regions, such as Japan, China, Southeast Asia, Australia, and the Pacific Islands [2,3]. Most plants in the genus *Vitex* are shrubs or arbors. [4]. Often used as a remedy to women’s health problems and other reproductive ailments, *Vitex* is also universally known as chaste tree. The plant got its name from the notion that it may help treat infertility and reduce libido. With respect to different sorts of *Vitex* species and depending on the country, they are also known by a variety of names, such as Nirgundi, Sambhalu, Gattiler, Arabian Lilac, Manjingzi, and Legundi [5,6,7,8]. Despite the different names, *Vitex* has been historically known and utilized by prehistoric civilization for various purposes, including treatment for reproductive disorders, gastrointestinal conditions, inflammatory diseases, and the ability to subdue several symptoms of psychiatric illnesses [9,10,11,12]. Nowadays, many studies are being conducted to investigate the biological properties of *Vitex* genus, as they are believed to contain a pool of metabolites with potential complementary therapeutic actions.

The available literature reveals some of the bioactivities exhibited by the *Vitex* genus are anti-inflammatory, analgesic, antihistamine, antimicrobial, antioxidant, and cytotoxic activities against various cancer cell lines [3]. For example, it was found that ethanolic extract from the fruit of *V*. *agnus-castus* L. indicated inhibition activity against D_2_ and opioid receptors [13]. The dopaminergic compounds are also beneficial for the treatment of premenstrual mastodynia and some other symptoms linked with premenstrual syndrome [14]. Apart from being known for its anti-inflammatory activities, *V*. *peduncularis* Wall. Ex Schauer is also being investigated for its antimicrobial properties. [15] reported that the methanolic extract from the stem and leaf of *V*. *peduncularis* Wall. Ex Schauer exhibited both antibacterial and antifungal activities against *Enterobacter aeurogens*, *Staphylococcus aureus*, *Candida*, and *Rhizopus* species, respectively. In addition, among five distinct species of *Vitex,* namely *V*. *trifolia* L., *V*. *negundo* L., *Vitex altissima* L. f., *V*. *peduncularis* Wall. Ex Schauer, and *Vitex diversifolia*, it was discovered that methanolic extract from the leaf of *V. peduncularis* Wall. Ex. Schauer contained the highest growth inhibition activity against all tested human pathogenic bacteria in the study [16]. Another *Vitex* genus known for its biological significance is *V*. *trifolia* L. When tested against carrageenan-induced paw oedema in rats, it was observed that the hydroalcoholic extract of *V*. *trifolia* exhibited anti-inflammatory properties [17]. A study group treated with the plant extract showed lower levels of mast cells, inflammatory mediators, and macrophages as compared to animals treated with control group indomethacin. *V*. *trifolia* L. extract is reported to express cytotoxic activity against several cell lines, including colon carcinoma, ovarian cancer, and cervix carcinoma cells, and they also found that the hexanic extract of the leaf managed to completely inhibit the growth of fungal pathogen *Fusarium* species [18]. *Vitex,* such as *V*. *negundo* L., is also studied for its analgesic effect. [19] discovered that after 1 h of therapy, a study group of rats treated with an aqueous extract of *V*. *negundo* L. leaf demonstrated considerable dose-dependent analgesic efficacy.

**Figure 1:**
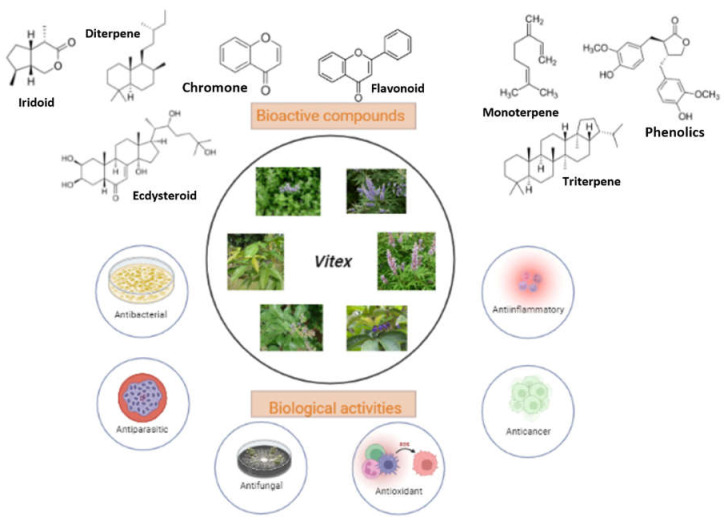
Bioactive secondary metabolites and biological activities of *Vitex* sp.

The *Vitex* genus has gained attention from the health sector market due to the pharmacological potentials contributed by the phytochemicals present in the plant matrix. They can be promoted as a variety of supplements to help improve and treat different kinds of illnesses or utilized as complementary medicine along with other standard treatment. At present, many researchers are interested in discovering new pharmacologically active compounds of *Vitex* plant. Different types of secondary metabolites are found from the *Vitex* genus, namely terpenes, steroids, flavonoids, lignans, and phenolic compounds [20]. Some of the bioactive compounds of *Vitex* noted to be responsible for its anti-inflammatory activity are iridoid and pedunculariside, where they were found to demonstrate preferential inhibition of COX-2 and little inhibitory effect on COX-1 [21,22]. Flavonoids extracted from *Vitex* also contributed to several pharmacological benefits of the plant, such as the antifungal activity, trypanocidal activity, and anti-filarial properties [23,24,25]. In addition, diterpenoid and triterpenoid are also some of the constituents found in the *Vitex* genus to exhibit anti-proliferative properties, cytotoxic activity, and demonstrated a dopaminergic effect [26,27,28,29]. Despite the various discoveries of *Vitex* metabolites and their biological effects, the preponderance of *Vitex*’s commercial product is used to treat menstrual problems, including inconsistent menstruation, mastodynia, and relieved symptoms associated with premenstrual syndrome [30]. For instance, Blackmores, Kordels, Thompson, Nature, and Solaray are some of the well-known supplement companies that have successfully commercialized *Vitex*-based products. Figure 1 provides a summary of *Vitex* species and highlights the main compounds and their biological activities that have been recorded. Previously, several review papers on the genus *Vitex* have been published, between 2005 and 2021, which cover specific species, such as *V*. *agnus*-*castus* L. [5,31,32], *V*. *negundo* L. [33,34], *V*. *trifolia* L. [35], and *V*. *rotundifolia* [36], and mostly discussed the ethnobotany and pharmacological activities. In terms of secondary metabolites, one review paper published in 2016 [5] only focused on terpene derivatives, while a review paper in 2021 [37] extensively discussed sesquiterpene. However, the information is lacking on the application of *Vitex* in human clinical trials, toxicology and safety, marketed products, and patents. Therefore, the purpose of this review is to offer an overview of the traditional medicinal uses, secondary metabolites, and pharmacological effects of secondary metabolites isolated from *Vitex* species that may be utilized as a reference for future research and use of the species. In addition, the application of *Vitex* in human clinical trials, toxicology and safety, marketed products, and patents will be included.

## 2. Methods of Literature Search

**Figure 2:**
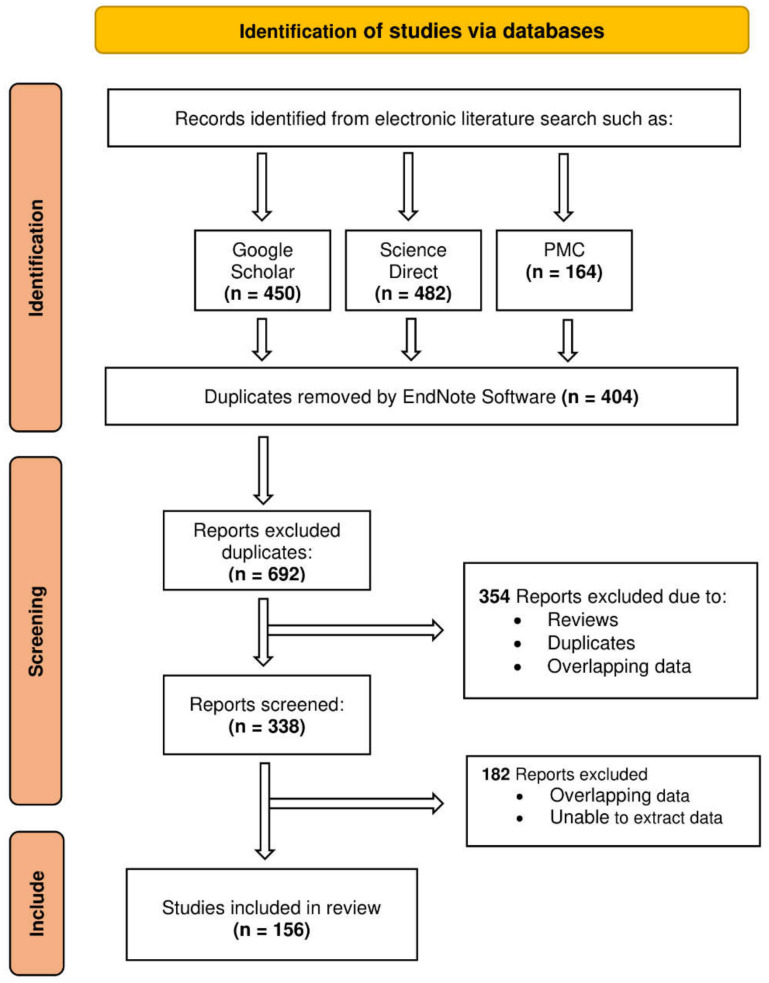
Prisma flow diagram of the study.

An exhaustive search of the published literature using a variety of books and online databases, such as Google Scholar, ScienceDirect, and PubMed Central, was carried out with the purpose of obtaining, compiling, and synthesizing information that is currently available on a variety of fundamental aspects pertaining to *Vitex* plants. This systematic review was conducted by adopting PRISMA (Preferred Reporting Items for Systematic Reviews and Meta-Analyses), as shown in Figure 2 below.

## 3. Traditional Medicinal Use of *Vitex* Species

The *Vitex* plant has been known as a medicinal plant and used as herbal medicine in the past, where it was recognized and noted in many health practices, including Ayurveda, Unani medicine, Chinese traditional medicine, Malay traditional medicine, European medicine, and ancient Greek medicine [6,10,12]. They mentioned the use of *Vitex* for a wide range of conditions, such as to treat women with reproductive disorders, improve body health after childbirth, suppress libido, treat skin problems, cure symptoms of gastrointestinal afflictions, reduce fever, heal rheumatism, and a lot more [10,12,38,39,40,41]. For example, *V. agnus-castus* L., *V*. *negundo* L., *V*. *peduncularis* Wall, and *V*. *trifolia* L. Ex Schauer are some of the *Vitex* species commonly used in the preparation of traditional remedies [3,12,42]. Many reports noted that the medicinal properties of *Vitex* came from different parts of the plant, mainly the leaves, fruits, and barks. They were made into paste, consumed as decoctions, smoked, and preserved as dried fruits.

### 3.1. Traditional Medicinal Uses of Vitex negundo L.

Locally known as Nirgundi or Sindhvar in India and the Philippines, *V. negundo* L. plant is widely planted along the road as a hedge plant and is utilized as traditional medicine to treat a variety of medical issues, some of which have been empirically verified. Traditionally, the plant is reported by many studies as an important medicinal plant, specifically in India, where they exhibit multifarious activities, including anti-inflammatory, analgesics, tonic, and antimicrobial properties. In India and Malaysia, the shoot, fruit, and leaf of Nirgundi plant are used to help women after childbirth where the juice of the shoot and fruit is utilized to increase milk lactation and the leaf was boiled in water for post-partum bath, which helps the mother’s recovery [39,40]. Different plant sections of *V*. *negundo* L., such as the root, bark, and flower, have also been used extensively in India and Pakistan as decoctions to treat gastrointestinal disorders, such as diarrhea, dysentery, flatulence, indigestion, and cholera [38,41]. Other than that, the leaves are exploited to relieve headache in Bangladesh, India, and Malaysia [43,44,45]. They are used to ease headache in a variety of ways, where, in Bangladesh, leaves are crushed and put into poultice, the Indians smoked the leaves, and in Malaysia, they stuffed pillows with the leaves. Apart from headache, the Nirgundi leaves are also popular in India, China, and Nepal to heal cough and sore throat where they are made into juice [42,46,47]. Meanwhile, the Singhalese used the plant to treat rheumatism by powdering the root to use it as tincture, as well as extracting juice from the leaves [48]. In a review study, it was noted that *V*. *negundo* L. is also used to cure several skin-related ailments among the Assamese [49]. For instance, the paste and juice are made from the leaves for topical application to skin and used orally to treat cellulitis and hives. The juice can also be utilized to cure carbuncle by mixing it with oil extracted from *Sesamum indicum*. In Ayurveda medicine, a decoction from the *V*. *negundo* L. leaf with *Piper nigrum* is effective to cure catarrhal fever and muffled hearing, while Unani medicine cited a mixture of *V*. *negundo* L. and sugarcane, which is taken orally to reduce swelling [12,50].

### 3.2. Traditional Medicinal Use of Vitex agnus-castus L.

*V*. *agnus-castus* L. has been historically known around the world by different names, including chaste tree, Sambhalu among the Urdu, Gattilier in French, and Sambha in Hindu [5]. The plant is popular due to its health benefits in the reproductive system where locals resort to the plant to help reduce sexual desire and it is used to treat female disorders linked with the reproductive system. It was reported that in ancient times, Roman wives made use of the aromatic leaves to reduce the libido of their husbands. In Latin, the locals produced beverages from *V*. *agnus-castus* L. seeds for the same reason as the Romans [51]. *V*. *agnus-castus* L. is also interchangeably called the pepper or berry of Monk because monks used to chew the berries, include the berries in their food, and placed them in their pockets to suppress their sexual desire in prehistoric days [52,53,54]. Athenian women also used the plant during the Thesmophoria festival to keep their chastity by covering their bed linen with the foul-smelling leaves [55]. Some other reports noted that the chaste tree has not only received support from European and North American traditional herbalists and practitioners to treat gynecological disorders, such as menstrual irregularities, infertility, and premenstrual syndrome, but also used to manage acne problems, digestive complaints, and act as a sedative [56,57]. European herbalists utilized chaste plant to stimulate the uterus and encourage menstruation among local women [55]. Iranian and Albanian traditional medicine also utilized the leaves and fruits of chaste plant to increase milk production for women after childbirth [58,59]. Other than that, it was reported that before the Common Era, Hippocrates recommended chaste tree for inflammation and injuries, and after four centuries, the Greek botanist Pedanius Dioscorides also suggested the same plant to increase lactation and reduce womb inflammation [10]. Due to its hot nature, the Greeks, Egyptians, and Romans took the seeds of chaste plant to dispel wind or relieve bowel flatulence, promote urine, and treat dropsy and splenic disease [10]. They also prepared antidotes for spiders and snakes bites from the same plant. In several regions of India and Pakistan, such as Loralai, Bori, and Barkhan, the locals used the plant to treat pains due to chills by taking baths in water boiled with the leaves [12]. During the middle age, Arabians, Salerno natives, and Anglo-Saxons recognized the *V*. *agnus*-*castus* L. plant as a remedy to treat symptoms of psychological illnesses. It was revealed that the fruit of chaste plant was sold at Arabian bazaars as a calming agent for hysteria [10]. The author also added that the locals mixed *V*. *agnus*-*castus* L. with other herbs to create a remedy to treat epilepsy and mental illness. The utilization of chaste plant in Chinese, Indian, or Ayurveda traditional medicine is not commonly employed for disease therapy.

### 3.3. Traditional Medicinal Uses of Vitex trifolia L.

Prevalently grown in tropical and subtropical regions, *V*. *trifolia* L. is a medicinal plant that can be found in China, India, Indonesia, Sri Lanka, Australia, and Singapore. It is called by different names all around the world, such as Arabian Lilac and Fructus Viticis in English, Jalanirgundi in Sanskrit, Nichinda in Hindi, and Manjingzi in Chinese [6,7]. In a review report, it was noted that *V*. *trifolia* L. was utilized traditionally to treat several different ailments, including joint pains, ringworm infection, leprosy, and skin rashes [60]. The author found that decoctions from the leaves of *V*. *trifolia* L. are given orally to ease joint and sciatica pains in Asian countries, while for the treatment of leprosy and skin rashes, the leaves can be ingested together with honey or applied topically. They also added that a mixture of crushed leaves and ghee (semifluid butter made from animal’s milk, usually cow or buffalo’s milk) is traditionally applied on the area infected with fungus. The mixture of *V*. *trifolia* L. leaves and honey has also been given in cases of intermittent fever with severe thirst and vomiting [12]. According to Ayurveda, the combination of Jalanirgundi leaves and other herbs is believed to help regulate the accumulation of pitta (In Ayurverda, they believe pitta represents heat, fire, and energy) in the blood and the root can be used to make tonic, act as expectorant and febrifuge [12,60]. In Chinese traditional medicine, the fruit of this plant is used to ease headache, migraine, common cold, eye pain, and in certain regions of China, it is applied as traditional medicine to treat specific cancers [61,62]. It was noted that pillows stuffed with the leaves of *V*. *trifolia* L. are effective to cure catarrh and headache [12]. Sambhalu is another name for the plant in Unani medicine and was described to be utilized with the purpose of decreasing libido [7]. In Papua New Guinea, the natives make use of the stem of *V*. *trifolia* L. to treat dysentery. The plant leaves are also used to manage dysentery in New Caledonia and the Samoans applied them topically to relieve sprained joint and rheumatic pain [11]. It was also reported that the leaves are utilized into medication in New Caledonia, Rotuma, and the Solomon Islands to alleviate headache by heating them and rubbing on the forehead or taken as an infusion [63]. In Tonga, they employed the plant to cure oral infections and inflammations [63].

### 3.4. Traditional Medicinal Uses of Vitex peduncularis Wall. Ex Schauer

Commonly known by different names in certain regions, *V*. *peduncularis* Wall. Ex Schauer can be discovered in Bangladesh, India, Vietnam, and Myanmar. The plant is also natively called Horina in Chittagong, Korobaong in Bengali, Chang Xu Jing in China, and Charaiygoda in Hindi [64]. The traditional medicine system used various portions of *V*. *peduncularis* Wall. Ex Schauer, including the bark, roots, and leaves, to cure various illnesses. In Bangladesh, different ethnic communities have employed Korobaong plant for different purposes. For example, the natives around the hill area of Khagrachari utilized the leaves and barks of the plant as a remedy for diabetes [64]. Meanwhile, the Chakma people made paste from the bark of the plant and topically used it for the treatment of jaundice and numbness of the face and eyes [64,65]. They also used the same paste and took it with water to treat urethritis [9]. It was reported that the barks, roots, and leaves of *V*. *peduncularis* Wall. Ex Schauer have been used as a folk remedy to heal blackwater fever and malaria [66,67]. Girach, Aminuddin, Siddiqui, and Khan (1994) noted the prescription of using *V*. *peduncularis* Wall. Ex Schauer to treat malaria fever where a few strands of leaves are heated in 1 L water up until the final volume is lowered to 250 mL, then filtrated, and it is consumed as two teaspoons twice a day. They also added that the stem bark of the plant is made into juice and served hot to treat malaria fever. The bark of the plant is also boiled and drunk to ease chest pain and treat joint ache [9]. Kirtikar and Basu (1935) noted that in Chota Nagpur, the locals exploited the *V*. *peduncularis* Wall. Ex Schauer plant’s bark for external application to treat pain in the chest. Table 1 displays the summary of traditional uses of different *Vitex* species for medicine.

## 4. Secondary Metabolites Isolated from *Vitex* and Their Biological Activities

### 4.1. Iridoids

Iridoids are cyclopentano[c]pyran monoterpenoids, which have been isolated from both terrestrial and marine organisms. Those mainly isolated from plants are glycosidic in nature. In terms of chemotaxonomy and biogenesis, iridoids are structurally associated with both terpenes and alkaloids [69]. Previous studies described the diverse bioactivities of these compounds, for cardiovascular, anti-inflammatory, antisplasmodic, anticancer, antiviral, anticholinesterase, antifungal, and antihepatotoxic effects [70].

**Figure 3:**
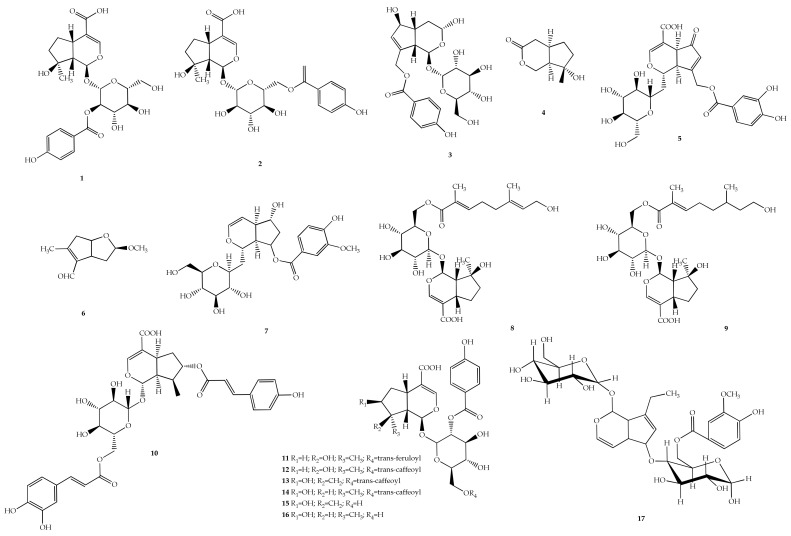

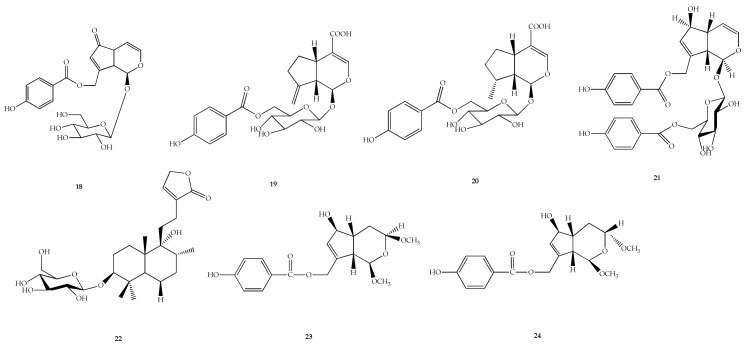
Chemical structures of iridoids **1**–**24** found in *Vitex* species.

*V*. *negundo* L. is the most widely studied species, producing five new iridoids. Two mussaenosidic acid derivatives, namely 2-*p*-hydroxybenzoyl mussaenosidic acid or known as negundoside (**1**) (Figure 3) and 6-*p*-hydroxybenzoyl mussaenosidic acid (**2**), were the first iridiods, which were isolated from the genus *Vitex* [71,72]. Compound **1** showed protective effects against carbon tetrachloride (CCl_4_)-induced toxicity in hepatocyte-derived carcinoma (HuH-7) cells and oxidative stress [40]. Another iridoid nishindaside (**3**) was isolated from *V*. *negundo* L. for the first time [73], lagundinin (**4**) [74] and 1,4a,5,7a-tetrahydro-1-D-glucosyl-7-(30,40-dihydroxybenzoyloxymethyl)-5-ketocy-clopenta[c]pyran-4-carboxylic acid (**5**) [75] were also discovered from the same species. Tarumal (**6**) was isolated from Brazilian *V. cymosa* Bertero ex Spreng [21]. *V*. *peduncularis* Wall. Ex Schauer yielded pedunculariside (**7**), which was shown to inhibit cyclooxygenase-2 (COX-2) with an IC_50_ value of 0.15 ± 0.21 mg/mL. However, it showed low cyclooxygenase-1 (COX-1) inhibition and no cytotoxicity against African green monkey kidney (Vero) cell lines was reported [22].

Three iridoids, namely agnucastoside A–C (**8**–**10**), were derived from *V. agnus-castus* L. with no activity in both antimicrobial and anticancer assays [76]. Another six iridoids, namely 6′-*O*-*trans*-feruloylnegundoside (**11**), 6′-*O*-*trans*-caffeoylnegundoside (**12**), 2′-*O*-*p*-hydroxybenzoyl-6′-*O*-*trans*-caffeoylgardoside (**13**), 2′-*O*-*p*-hydroxybenzoyl-6′-*O*-*trans*-caffeoyl-8-epiloganic acid (**14**), 2′-*O*-*p*-hydroxybenzoyl gardoside (**15**), and 2′-*O*-*p*-hydroxybenzoyl-8-epiloganic acid (**16**), were isolated from an ethyl acetate extract of *V. altissima* L.f. Compounds **12**–**14** exhibited promising antioxidant activity in both 1,1-diphenyl-2-picrylhydrazyl (DPPH) radical-scavenging and superoxide free-radical-scavenging assays [77]. A new iridoid metabolite, pinnatoside (**17**), was derived from *Vitex pinnata* L. as one of its minor compounds [78].

The methanol extract from flowers of *V. agnus-castus* L. produced new iridoid glycosides, namely agnusoside (**18**). Compound **18** has been identified to possess moderate anti-inflammatory activity using lipopolysaccharide-induced nitric oxide (NO) production [79]. Two new iridoid glycosides were found in *V. negundo* var. *heterophylla*, such as vitexnegheteroins K and L (**19** and **20**) [80] and 10-*p*-hydroxybenzoyl-6*β*-hydroxyiridoid 1-*O*-*β*-D-(6′-*O*-*p*-hydroxybenzoyl)glucopyranoside (**21**) [81]. The iridoid glycosides **19** and **20** exhibited weak antioxidant effects with IC_50_ value >20 µM and moderate inhibitory effects on *α*-glucosidase. Meanwhile, compound **21** did not inhibit NO production activity up to a concentration of 100 µM.

Furthermore, a new iridoid glycoside was found in *V*. *trifolia* L. producing (1*S*, 5*S*,6*R*,9*R*)-10-*O*-*p*-hydroxybenzoyl-5,6*β*-dihydroxy iridoid 1-*O*-*β*-D-glucopyranosidem (**22**) [82]. The isolated compound **22** was assessed for NO inhibitory activity tested on LPS-induced murine macrophage (RAW 264.7) cells to exhibit moderate inhibitory activity with an IC_50_ value of 90.05 μM.

*V. negundo* var. *heterophylla* is the most studied species to identify two new iridiod aglycones, namely ishindacin A (**23**) and isonishindacin A (**24**), which were identified for the first time from the plant [83]. Both compounds **23** and **24** showed a weak radical scavenging effect on stable free radicals, with percentage scavenging activities of 27.14% and 25.80%, respectively.

### 4.2. Diterpenoids

**Figure 4:**
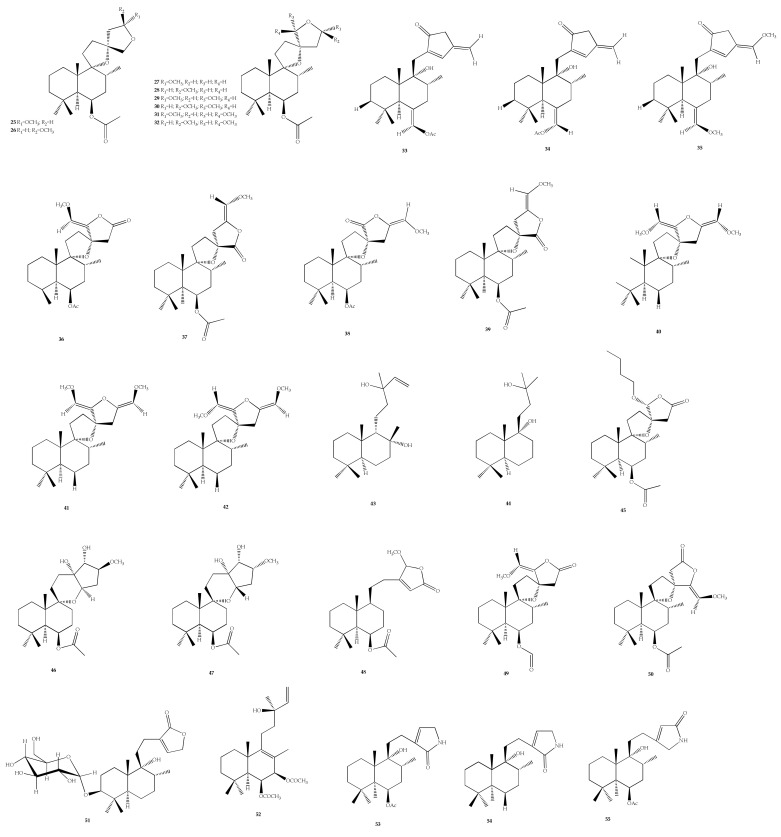

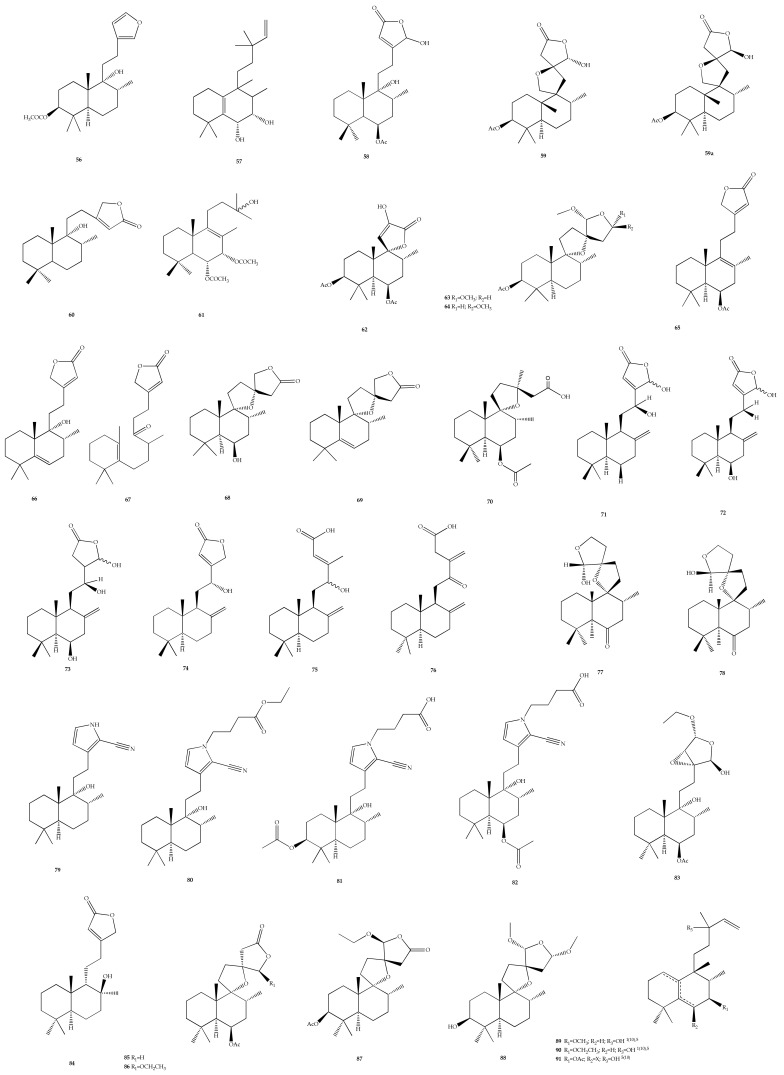

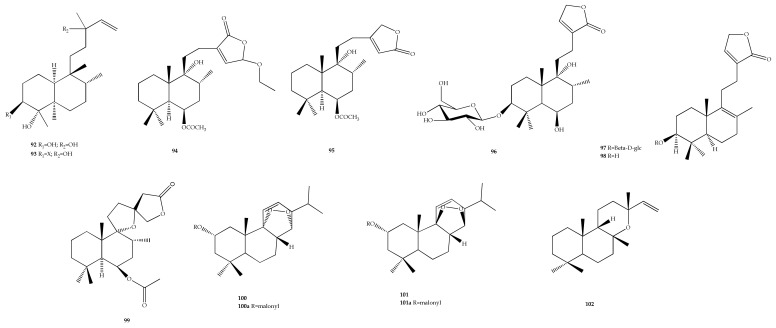
Chemical structures of diterpenoids **25**–**102** found in *Vitex* species.

Diterpene is a type of terpene that has twenty carbons and is biosynthetically derived from geranylgeraniol pyrophosphate. In the *Vitex* species, the labdane-type diterpenes are the most typical. In addition, abietane-, nor- and halimane-type diterpenes were also found in *Vitex* sp. (Figure 4). The group of Masateru Ono from Japan dedicated their research to finding new labdane diterpenes from the fruits of *V. rotundifolia* and *V. agnus*-*castus* L. Five journal papers have been published by this group, reporting the discovery of 27 labdane-type diterpenes, in which eight labdane-type diterpenes **25**–**32** were isolated from *V. rotundifolia*. Compound **32** was subjected to an antioxidant assay but did not exhibit any activities [84].

Moreover, ten labdane-type diterpene congeners **33**–**42** were isolated from the same species. Unfortunately, no bioactivity was recorded. Compounds **35**–**42** may be artefacts resulting from reactions with aldehydes during isolation work [85]. The isolation of eight more labdane diterpenes was achieved from the fruit of *V. agnus*-*castus* L., including viteagnusin C–H and J (**43**–**49**), viteagnuside I (**50**), and a labdane diterpene glucoside, namely viteagnuside A (**51**) [86,87,88].

Labdane diterpene 6*β*,7*β*-diacetoxy-13-hydroxy-labda-8,14-diene (**52**) was extracted from the hexane extract of *V. agnus*-*castus* L. fruits possessing strong affinity to the dopamine-D2-receptor with IC_50_ value of 15 μg/mL [27]. From the fruits of *V*. *agnus*-*castus* L., a novel nitrogen-containing labdane diterpene called vitexlactam A was isolated [89]. Another two labdane diterpene alkaloids, named vitexlactam B and C (**54** and **55**) have been isolated from *V. agnus*-*castus* L. along with compound **52**. Their cancer chemoprevention effect was tested and only compound **55** showed moderate results on NADP(H); quinone oxireductase type1 (QR1) induction activity [90].

Vitetrifolin H and I (**56** and **57**), two more labdane diterpenes, were isolated from the fruits of *V. trifolia* L. and both compounds inhibited cervical cancer (HeLa) cell proliferation, with IC_50_ values between 4 and 28 μM. Moreover, compound **57** was found to induce G0/G1 phase arrest and apoptosis of HeLa cells [29]. Viteagnusin I (**58**) was found from the fruits of *V. agnus*-*castus* L.; however, no activity was reported on opioid receptor assays DOR and MOR [91].

A mixture of two diastereomers of labdane diterpene, negundol (**59** and **59a**), which was isolated from *V*. *negundo* L. possessing antifungal activity, with MIC values in the range of 16–64 μg/mL [92]. Two new labdane diterpenoids, such as 6*α*,7*α*-diacetoxy-13-hydroxy-8(9),14-labdadien (**60**), and 9-hydroxy-13(14)-labden-15,16-olide (**61**), were found in the leaf extract of *V*. *trifolia* L. Compound **60** was found to be active against *Mycobacterium tuberculosis,* with an MIC value of 100 μg/mL [93].

In addition, norlabdane diterpene vitrifolin A (**62**) was also isolated from *V*. *trifolia* L. Linn. var. *simplicifolia* and has a moderate inhibitory effect against NO production in lipopolysaccaride-activated mouse macrophages [94]. Seven labdane diterpenes, including vitextrifolin A–G (**63**–**69**), were derived from the fruits of *V*. *trifolia* L.; however, they were found to be inactive against four human cancer cell lines (A549, HCT116, HL60, and ZR-75-30) [95].

Another derivative of labdane-type diterpenoids, known as vitrifolin B (**70**), was isolated from the fruits of V. Rotundifolia [96]. The leaf extract of Vitex vestita Wall. Ex Walp., which was collected from Machang, Kelantan, Malaysia, was screened for its antimicrobial activity, leading to the isolation of six labdane-type diterpenoids. The compounds are, namely 12-epivitexolide A (**71**), vitexolide B-C and E (**72**–**74**), vitexolin A and B (**75** and **76**) [26]. Compounds **71**–**73** exhibited similar infrared spectra due to the presence of an exomethylene group of α,β-unsaturated γ-lactone and free hydroxyl groups. Compounds **71**, **74,** and **76** exhibited moderate antibacterial and cytotoxic activities against human colorectal carcinoma (HCT-116) cells and human fetal lung fibroblast (MRC15) cells, while compounds **72**–**73** and **75** exhibited cytotoxic activities only against HCT-116 cancer cell lines. Two new diterpenoid compounds, known as chastol (**77**) and epichastol (**78**), were discovered from the dried fruits of *V. agnus-castus* L., also known as *Viticis fructus* [97].

*V*. *trifolia* L. also produced three diterpenoid compounds, including vitepyrroloids A−D (**79**–**82**), in which only compound **79** showed cytotoxic activity against a human nasopharyngeal carcinoma (CNE1) cell line with IC_50_ value of 8.7 μM [98]. Other compounds were six labdane-type (**83**–**88**), three halimanes (**89**–**91**), and two clerodanes (**92** and **93**) [99]. Compounds **90** and **91** showed moderate cytotoxicity in the micromolar range. Compound **90** also showed equipotent Topoisomerase-1 (Top1) inhibition to camptothecin (CPT), but this compound was the most cytotoxic against HCT 116 cells with the highest Top1 inhibition. Meanwhile, compound **93** showed equipotent Top1 inhibition to CPT.

In *V. rotundifolia*, two diterpenoids were found, namely viterotulin C (**94**) and vitexilactone D (**95**) [100]. Both compounds were evaluated for their inhibitory effects on the nuclear factor-kappa B (NF-κB) pathway in human embryonic kidney (HEK 293) cells. These compounds inhibited tumor necrosis factor-α (TNF-α) -induced NF-κB activation. The compounds also significantly inhibited NF-κB activation (*p* < 0.05, *p* < 0.01), with inhibition rates of 66.09 ± 11.49 and 57.30 ± 15.70, respectively. There was also a new diterpenoid found in *V*. *trifolia* L., named (3*S*,5*S*,6*S*,8*R*,9*R*,10*S*)-3,6,9-trihydroxy-13(14)-labdean-16,15-olide-3-*O*-*β*-D-gluco-pyranoside (**96**) [82]. The isolated compound **96** was evaluated for its inhibitory effect on NO production in LPS-induced RAW 264.7 macrophages; however, no inhibitory activity was reported up to a concentration value of 100 μM.

Three new labdane-type diterpenoids were discovered, of which two compounds, namely (3*S*,5*R*,10*S*)-3-[(*β*-D-glucopyranosyl)oxy]-labd-8,13-dien-16,15-olide (**97**) and (3*S*,5*R*,10*S*)-3-hydroxy-labd-8,13-dien-16,15-olide (**98**), were isolated from *V. negundo* var. *heterophylla* [101], while 9-epivitexnegundin (**99**) was also isolated from the same species [102]. Compunds **97** and **98** were reported to exhibit strong inhibitory activity against NO production, in which compound **98** was the strongest inhibitor, with an IC_50_ value of 15.8 ± 1.38 μM, while compound **97** had an IC_50_ value of 40.10 ± 1.30 μM. Compound **98** also showed significant inhibition of pro-inflammatory cytokine interleukin 1-beta and 6 (IL-1β and IL-6) levels and able to inhibit inducible nitric oxide synthase (iNOS), COX-2, and NF-κB signal pathways [101]. Meanwhile, compound **99** was evaluated for its antimicrobial activity but the activity was not mentioned [102]. Another study on the *V*. *negundo* L. leaves from Vietnam reported that compound **99** was inactive against severe myelogenous leukemia tumor (K562) cells with IC_50_ value > 100 µM [103].

Four diterpenoids were isolated from *V. rotundifolia*, named as abieta-11(12)-ene-9*α*,13*α*-endoperoxide (**100**) and a malonyl derivative **100a**, two derivatives of abieta-11(12)-ene-9β,13β-endoperoxide (**101** and **101a**) and 9*α*H-manoyl oxide (**102**) [104]. All compunds were tested for antimalarial activity on *Plasmodium falciparum,* in which compound **101** had the most activity with an IC_50_ value of 1.2 µM ± 0.5 µM among others. It is likely that compound **101** was 54-times more active than compound 100 due to the existence of aperoxy bridge.

### 4.3. Ecdysteroids

**Figure 5:**
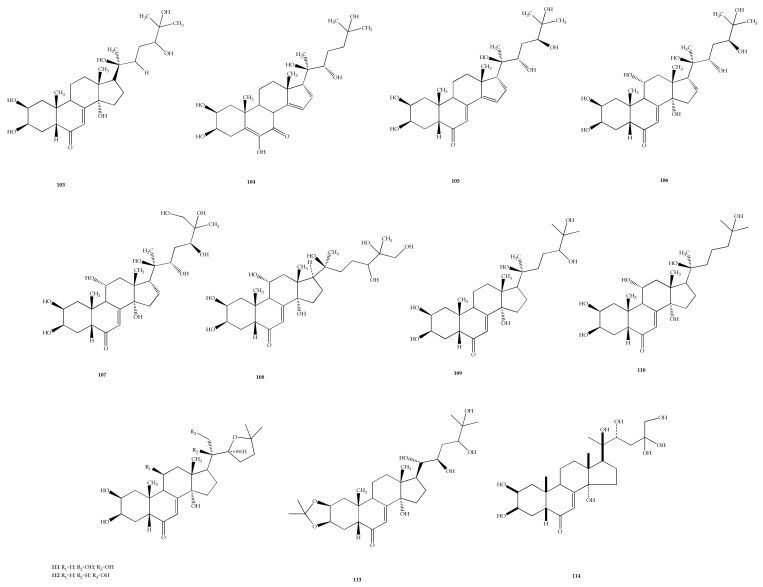
Chemical structures of ecdysteroids **103**–**114** found in *Vitex* species.

Ecdysteroids are hormones produced by insects that were initially believed to control molting and metamorphic processes. Nevertheless, their functions nowadays are more extensive than previously and it has been found that these hormones are produced at all development stages of an insect, commencing from newly laid eggs, embryonic stage, metamorphosis, reproduction, and diapause [105]. Plants also contain ecdysteroids (phytoecdysteroids) in large amounts as defense chemicals against phytophagous insects [106]. Compound ecdysteroids are described as in Figure 5.

In *Vitex* species, ecdysteroids are one of the common compounds reported to be able to act as chemotaxonomic markers in this genus [107]. Various ecdysteroids have been discovered from *Vitex*,, such as pinnatasterone (**103**) isolated from *V. pinnata* L. possessing weak activity against *Musca domestica* larvae [108]. Several ecdysteroids were also isolated from *V. canescens* Kurz, including canescensterone (**104**), 24-epiabutasterone (**105**), (24*R*)-11*α*,20,24-trihydroxyecdysone (**106**), and 11*α*,20,26-trihydroxyecdysone (**107**) [109,110,111].

Other ecdysteroids were 26-hydroxypinnatasterone (**108**) and 24-epi-pinnatasterone (**109**), which were isolated from *V. cymosa* Bertero ex Spreng. [112] and scabrasterone (**110**) discovered from *Vitex scabra* Wall. Ex Schauer, exhibiting very weak molting activity in the *Musca* bioassay [113]. *Vitex doniana* Sweet yielded 21-hydroxyshidasterone (**111**), 11*β*-hydroxy-20-deoxyshidasterone (**112**), and 2,3-acetonide-24-hydroxyecdysone (**113**), which showed anti-inflammatory activity in a rat paw oedema development assay at 100 mg/kg dose [114].

From *V. cienkowskii*, a new phytoecdysteroid was isolated from the stem bark of this plant, named 20,24-dihydroxy,24-hydroxymethylecdysone (**114**) [115]. Since ecdysteroids play an important role in chemical defence against non-adapted herbivores, this could explain the high abundance of this compound. It could be used as a new and additional characteristic compound parameter in the identification of compounds to reduce dereplication and false positives.

### 4.4. Flavonoids

**Figure 6:**
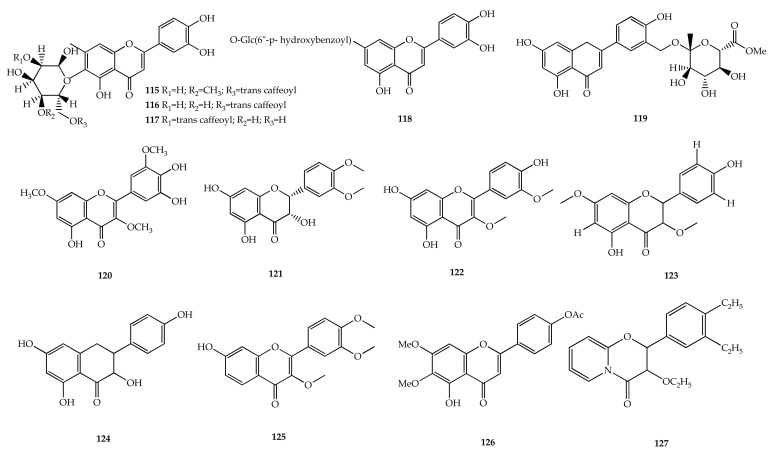
Chemical structures of flavonoids **115**–**127** found in *Vitex* species.

Flavonoids are a group of pigment compounds found abundantly in the plant kingdom [116]. These compounds are responsible for normal growth, development, and defense in plants. Flavonoid biosynthesis is formed via the shikimic acid and acylpolymalonate pathways [117]. Flavonoids isolated from *Vitex* were described as in Figure 6.

In the genus *Vitex*, a small number of flavonoids have been discovered. Four flavonoid glucosides isolated from *V. agnus-castus* L. exhibited cytotoxicity against lymphocytic leukemia (P388) cells. This included luteolin 6-C-(4″-methyl-6″-O-*trans*-caffeoylglucoside) (**115**), luteolin 6-C-(6″-*O*-*trans*-caffeoylglucoside) (**116**), luteolin 6-C-(2″-*O*-*trans*-caffeoylglucoside) (**117**), and luteolin 7-*O*-(6″-*p*-benzoylglucoside) (**118**), which were active against P388 cells with IC_50_ values of 7.6, 14, 56, and 70 μg/mL, respectively [118]. Vitegnoside (**119**) was found in *V*. *negundo* L. and exhibited antifungal activity on *Trichophyton mentagrophytes* and *Cryptococcus neoformans,* with an MIC value of 6.25 μg/mL [25].

A flavonol methyl ether, named vitecetin (**120**)**,** was found in *V. peduncularis* var. *cannobifolia* [119]. The in vitro antileishmanial activities of **120** on both *Leishmania donovani* promastigote and amastigote forms were assessed. The compound had potent antileishmanial activity, which is higher than sodium antimonygluconate (SAG), with IC_50_ values of 2.4 mM and 58.5 mM for promastigote and 0.93 mM and 36.2 mM for amastigotes, respectively. The compound was less toxic than SAG towards human leukemia monocytic (THP-1) cells, with 50% cytotoxic concentration (CC_50_) values of 123.7 mM and 364.3 mM, respectively.

The leaves of *V. Simplifocia* were collected from Nsukka, Nigeria, and further chemical isolation yielded five methylated constituents, namely 2-(5′-methoxyphenyl)-3,4′,5,7,8-trihydroxychroman-4-one (**121**), 2-(5′-methoxyphenyl)-4′,5,7-trihydroxy-3-methoxychromen-4-one (**122**), 2-(4′-hydroxyphenyl)-5-hydroxy-3,7-dimethoxy-chromen-4-one (**123**), 2-(4-hydroxyphenyl)-3,5,7-trihydroxychromen-4-one (**124**), and 2-(3′,4′-dimethoxyphenyl)-7-hydroxychromen-4-one (**125**) [23]. All flavonoids obtained from this species were tested for biological activities and exhibited moderate trypanocidal activity due to the increase in methylation of the hydroxyl group. Meanwhile, a flavonoid, which was isolated from the leaves of large evergreen tree *V. penduncularis* was 4′-acetoxy-5-hydroxy-6,7-dimethoxyflavone (**126**) [120].

In *V*. *negundo* L., there was one flavonoid assigned as 4,5-diethyl-3′-ethoxy-pyro-flavone (**127**) [24]. Compound **127** demonstrated significant in vitro antifilarial activity in a dose-dependent manner against adult *Setaria cervi* worms, as measured by worm motility and MTT reduction assays.

### 4.5. Miscellaneous

A class of phytonutrients called lignans is present in all plant species. [121]. Lignans have been known as minor constituents of plant varieties, where they are the building blocks for lignin formation in the plant cell walls [122]. There have been many studies reporting the association of lignans with human health benefits and disease acting as antioxidants, in an anti-inflammatory manner, and so on. A phenyldihydronaphthalene-type lignan named vitexdoin F (**128**) was found in the seeds of *V*. *negundo* L. [123]. The antioxidant activity on lignan was evaluated through DPPH radical-scavenging assays and exhibited obvious radical-scavenging effect on stable free radicals of DPPH. The compound also exhibited stronger activity than ascorbic acid.

The seeds of *V. negundo* var. *heterophylla* collected from Huludao, China, were discovered to contain three new phenylnaphthalene-type lignans, namely vitexnegheteroins E-G (**129**–**131**) and 9-hydroxysesamin (**132**) [124]. Compound **129** exhibited antioxidant and inhibitory activities on LPS-induced NO, while compound **130** exhibited moderate cytotoxic activity against human liver carcinoma (HepG2) cell lines. Meanwhile, compound **131** exhibited only antioxidant activity on the 2,2′-azino-bis(3-ethylbenzothiazoline-6-sulfonate) (ABTS) radical cation-scavenging activity. Two new phenylnaphthalene-type lignans, (3*R*,4*S*)-6-hydroxy4-(4-hydroxy-3-methoxyphenyl)-5,7-dimethoxy-3,4-dihydro-2-naphthaldehyde-3a-*O*-*β*-D-glucopyranoside (**133**) and 6,7,4′-trihydroxy-3′-methoxy-2,3-cycloligna-1,4-dien-2a,3a-olide (**134**), were isolated from the aerial parts of *V. negundo* var. *Heterophylla,* originating from Chaoyang city, China [125]. Then, two new lignans, namely 6-hydroxy-4-(4-hydroxy-3-methoxyphenyl)-3-acetoxymethyl-7-methoxy-3,4-dihydro-2-naphthaldehyde (**135**) and 3*α*-*O*-acetylvitedoin A (**136**), were isolated from the methanolic extract of *V. negundo* var. *cannabifolia* fruits using Sephadex LH-20 column chromatography, reverse-phase ODS gel, and silica gel [126]. The compounds were determined as artefacts as they had resulted from treatment with ethyl acetate during the fractionation and purification processes. In *V. Kwangsiensis* C. Pei, two new lignans were isolated from this plant and named as 6-hydroxy-4-(3,4-dimethoxyphenyl)-3-hydroxymethyl-7-methoxy-3,4-dihydro-2-naphthaldehyde or known as vitekwangin A (**137**) and 6-hydroxy-4-(3,4-dimethoxyphenyl)-3-hydroxymethyl-5-methoxy-3,4-dihydro-2-napthaldehyde (also known as vitekwangin B) (**138**) [127]. Compounds **137** and **138** were evaluated for their inhibitory activities on LPS-induced NO production using IC_50_ for RAW 264.7 is higher than 80 µM and both compounds had minor inhibitory effects on NO generation at lower doses.

Monoterpenoids are a type of terpenoids composed of two isoprene units, which are widely distributed in plants and used in pharmaceuticals and medicines [128]. Two new monoterpenoids were discovered from *V. negundo* var. *heterophylla* and named as (9*R*)-*O*-*β*-D-glucopyranosyloxy-2,5-megastigmen-4-one (**139**) and (3*S*,4*R*)-dihydroxy-7,8-dihydro-*β*-ionone4-*O*-*β*-D-glucopyranoside (**140** and **140a**). Both compounds demonstrated pronounced anti-inflammatory activity with IC_50_ > 100 μM, respectively [101].

Pentacyclic triterpenoids are widely found in the plant kingdom. Triterpenoids have been described as antiviral, anti-inflammatory, antitumor, and antimicrobial agents, while also acting as immunomodulator compounds [129]. In fact, some of these compunds are implicated in the resolution of immune diseases. Six new polyoxygenated triterpenoids, namely cannabifolins A−F (**141**–**146**), were found in *V. negundo* var. *cannabifolia* [130]. Compound **143** moderately inhibited NO production, with an IC_50_ value of 34.0 μM, while compounds **141**, **142**, **144** and **146** were inactive (<50% inhibition at 80 μM, the highest concentration tested). Moreover, compound **145** exhibited cytotoxicity on RAW 264.7 macrophages with cell viability less than 70% at 40 μM, and 3*β*-hydroxy-30-al-urs-12-en-28-oic acid (**147**) was isolated from the methanolic extract of *V. trifolia* var. *simplifocia* fruits, originating from the beach of Lang-Qi Island in Fuzhou, China [28]. Compound **147** was known to exhibit cytotoxic activity against leukemia (HL-60), gastric cancer (SGC-7901), pancreatic cancer (PANC-01), and esophageal carcinoma (Eca-109) in human cell lines. Compounds **129**–**131** and one triterpene compound vitexnegheteroin H (**148**) from *V. negundo* var. *heterophylla* showed inhibition of LPS-induced NO production in murine microglia (BV-2) cells [124]. Two new triterpenoids, such as 1*α*,3*β*-dihydroxybauer-7-en-28-oic acid (**149**) and 2*β*,3*β*,19*α*, 24-tetrahydroxy-23-norus-12-en-28-oic acid (**150**), were isolated from the leaves of *V. doniana* Sweet originated from farm land in Basawa village, Kaduna state [131].

The methanolic extract of *V. negundo* var. *heterophylla* seeds, which were collected from Huludao, Liaoning Province, China, produced eight new phenolic glucosides, namely vitexnegheteroins A-D (**151**–**154**), methyl (6-*O*-4-hydroxybenzoyl)-*α*-D-glucopyranoside (**155**), breynioside A (**156**), 1,6-di-*O*-4-hydroxybenzoyl-*β*-D-glucopyranoside (**157**), and dunnianoside D (**158**) [132]. These compounds showed antioxidant activity via ABTS radical scavenging assay and inhibitory activity on LPS-stimulated NO production. The in vitro bioactivities of these compounds were not comparable to actual in vivo beneficial effects, indicating that further studies on the in vivo bioactivities of the compounds should be conducted to provide real biological significance in future. Furthermore, a new phenolic glycoside, named 12-hydroxyjasmonic acid (6-*O*-caffeoyl)glucoside or known as vitexnegheteroin M (**159**), was isolated from the leaves of *V. negundo* var*. heterophylla*. The inhibitory effects on LPS-induced NO production in murine microglial cell BV-2 cells of **159** exhibited poor inhibitory effects on LPS-stimulated NO production (IC_50_ > 100 µM) and no cytotoxic effect on BV-2 cells [133].

**Figure 7:**
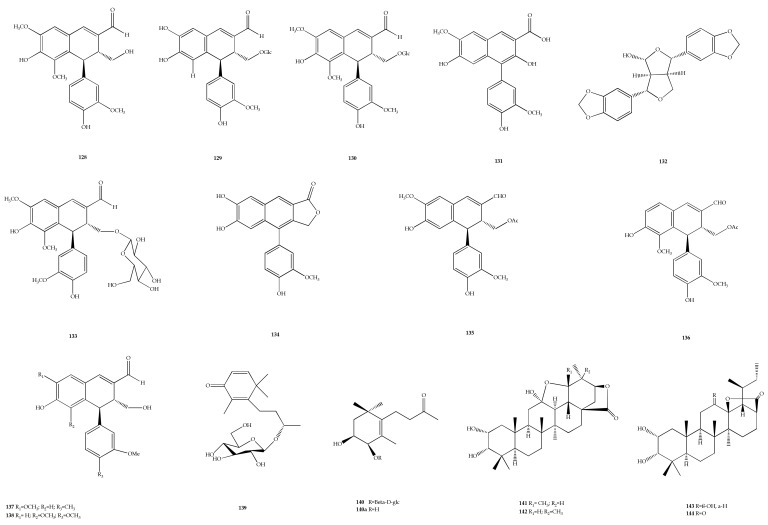

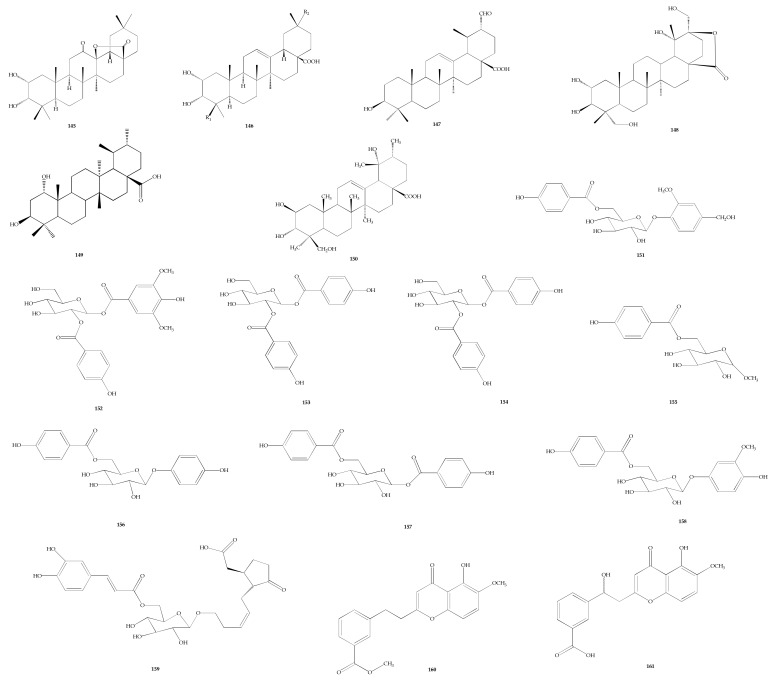
Chemical structures of compounds **128**–**161** found in *Vitex* species.

Chromone derivatives are abundant in nature and they are beneficial for a wide range of pharmacological activities, such as anticancer, antibacterial, antifungal, antioxidant, antiulcers, anti-HIV, immunostimulators, biocidal, anti-inflammatory, wound healing, and immune-stimulatory [134]. Two new chromone derivatives were found in *V*. *negundo* L., identified as methyl 3-(2-(5-hydroxy-6-methoxy-4-oxo-4*H*-chromen-2-yl)ethylbenzoate (**160**) and 3-(1-hydroxy-2-(5-hydroxy-6-methoxy-4-oxo-4*H*-chromen-2-yl)ethylbenzoic acid (**161**) [135]. In mice, these isolated compounds were examined for antinociceptive action in an abdomen constriction assay with acetic acid and paw oedema assay with carrageenan showing anti-inflammatory activity. They were successful in reducing nociception and inflammation, and *V*. *negundo* L. could be utilised as a source of antinociceptive and anti-inflammatory compounds. All compounds were described as in Figure 7.

Table 2 provides a summary of compounds isolated from various species of *Vitex* and their biological activities.

## 5. Health-Promoting Activities of *Vitex* in Humans, with Particular Regard to Clinical Trials

*V*. *agnus castus* L. is the only species in *Vitex* that has undergone clinical trials. The European Medicines Agency reported a total of 28 clinical trials related to the utilization of *V. agnus*-*castus* L. as a treatment for premenstrual syndrome (PMS), mastalgia, luteal insufficiency, menstrual bleeding disorders, amenorrhea, and menorrhagia [136]. From the 28 studies, there were 20 clinical trials to observe *V. agnus*-*castus* L.’s effect on premenstrual syndrome. One of the studies investigated the effect of *V. agnus*-*castus* L. on 50 women suffering from PMS [137]. Over the course of three menstrual cycles, the women received treatment with one tablet (20 mg native extract) each day and were given the Mood Disorder Questionnaire (MDQ) to rate 47 symptoms for self-assessment. A total of seven participants withdrew from the trials; six of them withdrew due to circumstances unrelated to the treatment and one person experienced headache and tiredness for the first 4 days of treatment. The remaining 43 participants completed eight menstrual cycle protocols (two baseline, three treatment, and three post-treatment) and 20 individuals reported 37 adverse effects throughout 344 menstrual cycles in total observed in the study. Acne (*n* = 7) was the most common occurrence, followed by six cases of headache (*n* = 6), five cases of spotting (*n* = 5), and five cases of gastrointestinal problems (*n* = 5). These occurrences are mostly common in PMS patients. For women with premenstrual syndrome, the effectiveness and tolerance of the agnus castus fruit (*V. agnus castus* L. extract Ze 440) were also evaluated in comparison to the placebo [138]. This three-menstrual cycle, placebo-controlled, double-blind trial included 170 women having premenstrual syndrome (active 86; placebo 84), with the same mean number for age, cycle length, and duration of menses. The VAC group (*n* = 86) consumed one *V. agnus*-*castus* L. fruit extract (Ze 440 20 mg) capsule once a day (made by Zeller AG; Romanshorn, Switzerland; 60 percent ethanol (m/m) extract ratio 6–12/1, standardized for casticin). The active group experienced fewer premenstrual symptoms in comparison to the placebo group. Another study on the therapeutic effect of *V. agnus*-*castus* L. on 128 women (mean age of 31 ± 4 years old) with premenstrual syndrome was conducted as a randomized and placebo-controlled trial [139]. The subjects were split into two groups: 62 were randomly assigned to receive 40 drops of *V. agnus*-*castus* L. extract daily and 66 were assigned to receive a similar placebo, commencing 6 days before menses. The patients were then assigned to complete self-assessment questionnaires prior to the trial and again, after six cycles of menstruation. A visual analogue scale (VAS) that ranged from 0 (asymptomatic) to 10 (intolerable) was used to grade each item. Before and after the study, the active and placebo groups’ rank of variables differed significantly (*p* < 0.0001) and similar differences were shown when *V. agnus-castus* L. was used compared to the placebo (*p* < 0.0001).

The European Medicines Agency also reported two clinical trials for the treatment of breast pain called mastalgia. There were 114 premenopausal participants in total aged 40 years old and younger with cyclic mastalgia included in a study [140]. The participants were administered 40 mg of the fruit of *V. agnus*-*castus* L. (group 1) and flurbiprofen (group 2). The authors concluded that both treatments show a significant reduction in the symptoms of mastalgia with no harmful effects. A randomized and controlled trial was then conducted to observe the effect of two treatment types, *V. agnus*-*castus* L. and flaxseed, on patients with cyclic mastalgia [141]. Block randomization was used to divide a total of 159 women into three groups, each with a size of 53 women. The group includes group 1 treated with 25 g flaxseed powder daily and *V. agnus*-*castus* L. placebo, group 2 treated with 3.2–4.8 mg *V. agnus*-*castus* L. capsules and flaxseed placebo, and a control group, which received both placebo treatments. In the first and second months, the breast pain intensity scores decreased significantly for both *V. agnus*-*castus* L. and flaxseed groups in comparison to the placebo group and the flaxseed group alone showed some adverse effects, such as dysentery symptoms and nausea.

Luteal insufficiency is a menstrual-cycle endocrine condition characterized by a reduced blood progesterone level and a shorter pregestational stage. The effectiveness of a 20 mg daily dose of *V. agnus*-*castus* L. extract on 52 women with luteal phase disorders, studied in randomized, placebo-controlled research [142]. Blood samples were taken at 5–8 and 20 days of menstruation cycle pre-treatment and after 3 months post-treatment. Then, by observing the release of prolactin, hyperprolactinemia was examined 15 min and 30 min after the intravenous injection of 200 µg Thyrotropin-releasing hormone (TRH). After 3 months, 37 full case reports (*n* = 20 for the placebo, *n* = 17 for the verum) show that the prolactin release reduced, luteal phase length normalized, and the luteal progesterone production deficit was eliminated in the verum group. The 17β-estradiol was shown to increase significantly in the luteal phase of treated patients.

There are no reported clinical trials on postmenstrual effect by the European Medicine Agency but there are recent studies that include postmenstrual syndrome in the observation. A report shows that the consumption of *V. agnus-castus* L. extracts can reduce menopausal symptoms, such as anxiety and vasomotor [143]. The researchers divided the patients into two placebo groups and a group treated with 30 mg *Vitex* and placebo medications for 8 weeks and then the Greene Questionnaire was used to assess the symptoms of menopause, before and after an eight-week intervention [144]. The results show that the group that consumed *V. agnus*-*castus* L. had less vasomotor dysfunction, anxiety, and overall menopausal disorder after the intervention. Recently, a randomized clinical trial was conducted on 89 postmenopausal women (mean age of 55.83 ± 3.63 years old) using random permuted blocks with a block size of three in the three groups [145]. The groups include *V. agnus*-*castus* L. group (3.2–4.8 mg/q8h), *Salvia officinalis* group (100 mg/q8h), and placebo group for three months. The author compared the postmenopausal women according to low-density lipoprotein (LDL), triglycerides (TG), and high-density lipoprotein (HDL), before and after the intervention. The *V. agnus*-*castus* L. extracts were able to lower the cholesterol (TG and LDL) and increased HDL compared with the placebo group.

## 6. Toxicology and Safety

It is crucial to study *Vitex*’s safety in humans as a potential medication drug. Numerous studies on *Vitex*’s safety have been carried out thus far. *V. agnus*-*castus* L.’s effects were reported to be minimal [31]. Nausea, minor gastrointestinal issues, exhaustion, menstruation difficulties, acne, dry mouth, erythematous rash, and pruritus are the most commonly reported side effects [31,146]. Concerning the consumption of *V. agnus*-*castus* L. while pregnant and nursing, there is still a lack of evidence based on theory, expert opinion, and in vitro research. There are studies to see the effects of *V. agnus*-*castus* L. but no evidence has shown that *V. agnus*-*castus* L. may have effects on estrogenic and progesterone activity [147,148]. As for lactation, there are different opinions on whether *V. agnus*-*castus* L. stimulates or lowers breastfeeding in lactation. Mixed findings were found to investigate *V. agnus-castus L.*’s effects on lactation. The compendia on herbal medicine and a plant monograph reported that *V. agnus*-*castus* L. elevates lactation [149,150] but there was also a report showing that *V. agnus*-*castus* L. decreases lactation due to the suppression in producing prolactin [151].

## 7. Marketed Products

According to a recent study, *Vitex* could be used to make pharmaceuticals for treating diseases, such as diabetes and many female conditions, for example, menstrual disorders and menopause [136]. This leads to *Vitex* being used in a variety of commercially available products, as shown in Table 3. One of the products available in the pharmaceutical market is Thompson’s-One-A-Day Vitex, manufactured by Thompsons Nutrition. It consists of 1500 mg 60 capsules that are suggested to be consumed as one capsule daily, which may help to relieve PMS and regulate the menstrual cycle. The *V. agnus*-*castus* L. extract formulation also helps to reduce PMS symptoms, including crankiness, breast pain, bloating, and the retention of fluid. Another product that used *V. agnus*-*castus* L. as an active ingredient is from nature, namely Nature’s Answer *V. agnus*-*castus* L. It is suggested to be used as a supplement taken for diet and recommended to take one capsule per day (40 mg/capsule) with food or water. This product helps to support a woman’s hormonal balance. Solaray Vitex (Chaste tree) consists of 400 mg 100 Vegetable Capsules, which are suggested to be taken in one capsule twice daily with food or water. Blackmores *V. agnus*-*castus* L. is a traditional Western herbal medicine that helps managed premenstrual symptoms and gives support to a healthy hormonal balance. The capsule is suggested to be taken one tablet three times a day before consuming any meals. Kordel’s Vitex is also traditionally used to manage premenstrual pain and regulate menstruation by taking 1–2 capsules daily, preferably before breakfast.

## 8. Patents

*V. agnus*-*castus* L., commonly known as chaste tree or chaste berry, is an herbal plant often used for various medicinal purposes. In pharmacology, *V. agnus*-*castus* L. was reported to have antibacterial, anti-inflammatory, anti-fungal, anti-microbial, antioxidant, and anticancer effects [152]. Four patents for the *Vitex* genus were recorded, most of which employ the *V. agnus*-*castus* L. species. The *Vitex* extract was used as a medicament to cure movement disorders, while other patents utilized the genus as a supplemental product to treat health issues that primarily affected women. Table 4 lists the patents available for *Vitex* genus and its health application.

## 9. Conclusions and Future Research Prospective

The objective of this review was to explore the composition of chemicals, biological, human clinical trials, toxicology and safety, marketed products, and patents of the genus *Vitex*. An extensive assessment of the literature reveals that sporadic information on 15 *Vitex* species has been evaluated for its pharmacological activity among other species of the *Vitex* genus. *V*. *negundo* L., *V*. *trifolia* L., *V. agnus*-*castus* L., and *V*. *peduncularis* Wall. Ex Schauer are some of the species that have been extensively utilized by prehistoric civilization and referenced in many traditional medicine systems, including Ayurveda, Unani, ancient Greek medicine, Chinese traditional medicine, and many others. They have been utilized for hundreds of years as a therapy for a variety of ailments, including premenstrual syndrome, inflammation, cancers, diabetes, skin-related diseases, and gastrointestinal conditions in the form of decoctions, pastes, and dried fruits.

Various studies have also found that the genus has intriguing pharmacological properties, which can be linked mostly due to its chemical components. The medicinal properties of the tree have piqued the interest of the health industry, as they can be commercialized as a range of supplement items as well as indicating health benefits of various parts and species of *Vitex*. Bioactive compounds present in *Vitex* plant matrix, such as iridoids, diterpenoids, ecdysteroids, and flavonoids, are reported to possess antibacterial, anti-inflammatory, antifungal, antioxidant, and anticancer properties, providing a scientific foundation for the use of genus *Vitex* as a valuable natural resource for developing new medicines and employed as disease treatment. Among various species of *Vitex*, *V. agnus*-*castus* L. is the only *Vitex* species that has been subjected to scientific testing and is utilized in herbal formulations that are sold as supplements in the market. Traditionally used to aid female disorders in the past, it can be observed that recent health application was also dedicated for the treatment of various health problems linked to female conditions. For instance, Hoberg et al. (2000) discovered that diterpenoid molecules isolated from *V. agnus*-*castus* L. demonstrated substantial dopaminergic activity, which might be a key player in treating female disorders. The *Vitex* species was also well known for its traditional uses in preventing cancer. This is corroborated by the results of many studies, which discovered that bioactive compounds, such as lignan and diterpenoid, exhibited significant cytotoxic activity and induced apoptosis events when observed in comparison to several cancer cell lines [26,29,98,99,124,127]. However, additional research is required to demonstrate the molecule linked to its conventional utilization in the past.

Despite the application of *Vitex* plant as supplements to treat menstrual conditions, the study found that few preliminary pharmacological reports were accessible discussing the phytochemicals accountable to the biological aspect of the plant. Some other potential medicinal actions of the *Vitex* genus that need to be explored include its effect on the endocrine system, antitumor properties, antimicrobial activity, antioxidant, and anti-inflammatory actions as most of the phytochemicals of the plant are constituents of flavonoids, iridoids, and diterpenoid components. Furthermore, further analyses should be performed through in vivo models to assess the potential of bioactive compounds expressed in different types of *Vitex* species. In addition, the application of system biology, such as metabolomics, is needed to unravel the secondary metabolites of *Vitex* extracts involved in various biological actions. Overall, the present review of *Vitex* pharmacological qualities and its chemical compounds will provide prospective knowledge on the current studies and identify areas that are opened for further research, so that issues related with its pharmacological aspects can be resolved. Therefore, on that basis, critical development of *Vitex* studies need to be carried out to improve the utilization of *Vitex* species as functional foods and pharmaceutical formulations with respect to their biological actions and availability in nature.

## Figures and Tables

**Table 1:** Summary of traditional applications of various *Vitex* species in medicine.

*Vitex* Species	Traditional Uses	Plant Parts Used	References
*Vitex negundo* L.	Increase lactation	Shoot, fruit	[39,40]
	Post-partum bath	Leaf	[39,40]
	Diarrhea, dysentery, flatulence, indigestion, and cholera	Root, bark, flower	[38,50]
	Headache	Leaf	[43,44,45]
	Cough, sore throat	Leaf	[42,46,47]
	Rheumatism	Root, leaf	[48]
	Hives, cellulitis, carbuncle	Leaf	[49]
	Catarrhal fever, muffled hearing	Leaf	[12,50]
*Vitex agnus-castus* L.	Decrease sexual drive	Leaf, seed, fruit	[51,52,53,54,55]
	Menstrual irregularities, infertility, premenstrual syndrome, acne, digestive complaint, sedative	-	[55,56,57]
	Increase lactation	Leaf, fruit	[58,59]
	Inflammation, injury, snake and spider antivenom, epilepsy, madness, insanity	-	[10]
	Flatulence, urination, dropsy, splenic disease	Seed	[10]
	Pain due to chills	Leaf	[12]
	Calming agent for hysteria	Fruit	[10]
*Vitex trifolia* L.	Joint pain, ringworm, leprosy, skin rashes	Leaf	[60]
	Intermittent fever, catarrh, headache	Leaf	[12]
	Pitta in blood	Leaf	[12,60]
	Tonic, expectorant, febrifuge	Root	[12,60]
	Headache, migraine, common fever, eye pain, cancer	Fruit	[61,62]
	Decrease sexual drive	-	[7]
	Dysentery	Stem, leaf	[11]
	Rheumatism, sprained joint	Leaf	[11]
	Headache	Leaf	[63]
	Oral infection, inflammation	-	[63]
*Vitex peduncularis* Wall. Ex Schauer	Diabetes	Leaf, bark	[64]
	Jaundice, numbness of face and eyes, urethritis	Bark	[9,64,65]
	Blackwater fever, malaria	Bark, root, leaf	[66,67,68]
	Chest pain, joint pain	Bark	[9,12]

**Table 2:** Summary of different compounds isolated from various *Vitex* species and their biological activities.

Compound Names	Structural Type	Biological Activities	*Vitex* Species	References
Negundoside (**1**) 6-*p*-hydroxybenzoyl mussaenosidic acid (**2**)	Iridoids	Showed protective effects against CCl_4_-induced toxicity in HuH-7 cells and oxidative stress.	*Vitex* sp.	[71,72]
Nishindaside (**3**)	Iridoids	-	*V*. *negundo* L.	[73]
Lagundinin (**4**)	Iridoids	-	*V*. *negundo* L.	[74]
Carboxylic acid derivative (**5**)	Iridoids	-	*V*. *negundo* L.	[75]
Tarumal (**6**)	Iridoids	Inhibited COX-2 with IC_50_ value of 0.15 ± 0.21 mg/mL. However, it only showed low inhibition of COX-1 and no cytotoxicity against African green monkey kidney (Vero) cell lines.	*V. cymosa* Bertero ex Spreng.	[21]
Pedunculariside (**7**)	Iridoids	-	*V*. *peduncularis* Wall. Ex Schauer	[22]
Agnucastoside A-C (**8**–**10**)	Iridoids	All three compounds showed no antimicrobial and anticancer activities.	*V. agnus-castus* L.	[76]
6′-*O*-*trans*-feruloylnegundoside (**11**) 6′-*O*-*trans*-caffeoylnegundoside (**12**) 2′-*O*-*p*-hydroxybenzoyl-6′-*O*-*trans*-caffeoylgardoside (**13**) 2′-*O*-*p*-hydroxybenzoyl-6′-*O*-*trans*-caffeoyl-8-epiloganic acid (**14**) 2′-*O*-*p*-hydroxybenzoylgardoside (**15**) 2′-*O*-*p*-hydroxybenzoyl-8-epiloganic acid (**16**)	Iridoids	Compounds **12**–**14** showed promising antioxidant activity in DPPH-radical-scavenging and superoxide free-radical-scavenging assays.	*V. altissima* L.f.	[77]
Pinnatoside (**17**)	Iridoid	-	*V. pinnata* L.	[78]
Agnusoside (**18**)	Iridoid	Exhibited moderate anti-inflammatory activity using NO production induced by lipopolysaccharide.	*V. agnus-castus* L.	[79]
Vitexnegheteroins K and L (**19** & **20**)	Iridoid glycoside	Iridoid glycosides **19**–**20** exhibited weaker antioxidant effects with IC_50_ values >20 µM.	*V. negundo* var*. heterophylla*	[80]
10-*p*-hydroxybenzoyl-6*β*-hydroxyiridoid 1-*O*-*β*-D-(6′-*O*-*p*-hydroxybenzoyl) glucopyranoside (**21**)	Iridoid glycoside	No inhibitory activity on NO production.	*V. negundo* var. *heterophylla*	[81]
(1*S*, 5*S*,6*R*,9*R*)-10-*O*-*p*-hydroxybenzoyl-5,6*β*-dihydroxy iridoid 1-*O*-*β*-D-glucopyranosidem (**22**)	Iridoid glycoside	IC50 value of 90.05 M and moderate inhibitory activities on NO production with LPS-induced RAW 264.7 macrophages.	*V*. *trifolia* L.	[82]
Nishindacin A (**23**) Isonishindacin A (**24**)	Iridoids	Compounds **23** & **24** showed weak radical-scavenging effects on stable free radical, with scavenging activity (%) of 27.14% and 25.80%, respectively.	*V. negundo* var. *heterophylla*	[83]
Labdane-type diterpenes **25**–**32**	Diterpenoids	No antioxidant activity.	*V. rotundifolia*	[84]
Labdane-type diterpene congeners **33**–**42**	Diterpenoids	No bioactivity was demonstrated. Compounds **35**–**42** could be artefacts resulting from the reactions with aldehyde during isolation process.	*V. rotundifolia*	[85]
Viteagnusin C-H and J (**43**–**49**) Viteagnuside I (**50**) Viteagnuside A (**51**)	Diterpenoids	-	*V. agnus*-*castus* L.	[86,87,88]
Labdane diterpene 6*β*,7*β*-diacetoxy-13-hydroxy-labda-8,14-diene (**52**)	Diterpenoid	It showed strong affinity to the dopamine-D2-receptor with IC_50_ value of 15 μg/mL.	*V. agnus*-*castus* L.	[27]
Vitexlactam A (**53**)	Diterpenoid	-	*V. agnus*-*castus* L.	[89]
Vitexlactam B and C (**54**–**55**)	Diterpenoids	Compound **55** showed moderate result on NADP(H); quinone oxireductase type1 (QR1) induction activity	*V. agnus*-*castus* L.	[90]
Vitetrifolin H and I (**56** & **57**)	Diterpenoids	Both compounds inhibited HeLa cell proliferation with IC_50_ between 4–28 μM. Compound **57** was also found to induce G_0_/G_1_ phase arrest and apoptosis of HeLa cells.	*V. trifolia* L.	[29]
Viteagnusin I (**58**)	Diterpenoid	No activity was shown on opioid receptor assays DOR and MOR.	*V. agnus*-*castus* L.	[91]
Two diastereomers of negundol (**59** and **59a**)	Diterpenoids	Exhibited antifungal activity with MIC values in the range of 16–64 μg/mL.	*V*. *negundo* L.	[92]
6*α*,7*α*-diacetoxy-13-hydroxy-8(9),14-labdadien (**60**) 9-hydroxy-13(14)-labden-15,16-olide (**61**)	Diterpenoids	Active against *M. tuberculosis* with MIC value of 100 μg/mL.	*V*. *trifolia* L.	[93]
Vitrifolin A (**62**)	Diterpenoid	Exhibited moderate inhibitory effect against NO production in lipopolysaccaride-activated mouse macrophages.	*V. trifolia* var. *simplicifolia*	[94]
Vitextrifolin A-G (**63**–**69**)	Diterpenoids	They were inactive against A549, HCT116, HL60, and ZR-75–30 cell lines.	*V*. *trifolia* L.	[95]
Vitrifolin B (**70**)	Diterpenoid	-	*V. rotundifolia*	[96]
12-epivitexolide A (**71**)	Diterpenoid	Exhibited moderate antibacterial and cytotoxic activity on the HCT-116 and MRC15 cell lines.	*V. vestita* Wall. Ex Walp.	[26]
Vitexolides B and C (**72** & **73**)	Diterpenoid	Exhibited cytotoxic activity on the HCT-116 cell lines.	*V. vestita* Wall. Ex Walp.	[26]
Vitexolides E (**74**)	Diterpenoid	Exhibited moderate antibacterial and cytotoxic activity on the HCT-116 and MRC15 cell lines.	*V. vestita* Wall. Ex Walp.	[26]
Vitexolin A (**75**)	Diterpenoid	Exhibited cytotoxic activity on the HCT-116 cell lines.	*V. vestita* Wall. Ex Walp.	[26]
Vitexolin B (**76**)	Diterpenoid	Exhibited moderate antibacterial and cytotoxic activity on the HCT-116 and MRC15 cell lines.	*V. vestita* Wall. Ex Walp.	[26]
Chastol (**77**)	Diterpenoid	-	*V. agnus*-*castus* L.	[97]
Epichastol (**78**)	Diterpenoid	-	*V. agnus*-*castus* L.	[97]
Vitepyrroloids A−D (**79**–**82**)	Diterpenoids	Exhibited cytotoxic activity against CNE1 cells with IC_50_ value of 8.7 μM.	*V*. *trifolia* L.	[98]
Labdane-types (**83**–**88**)	Diterpenoids	-	*V*. *trifolia* L.	[99]
Halimane (**89**–**91**)	Diterpenoids	Compounds **90** and **91** showed moderate cytotoxicity at micromolar range. Compound **90** showed equipotent Top1 inhibition to CPT and the most cytotoxic against HCT 116 cells with the most highest Top1 inhibition.	*V*. *trifolia* L.	[99]
Clerodane (**92**–**93**)	Diterpenoids	Compounds **93** showed equipotent Top1 inhibition to CPT.	*V*. *trifolia* L.	[99]
Viterotulin C (**94**) Vitexilactone D (**95**)	Diterpenoids	Both compounds significantly inhibited NF-κB activation (*p* < 0.05, *p* < 0.01) with inhibition rates of 66.09 ± 11.49 and 57.30 ± 15.70, respectively.	*V. rotundifolia*	[100]
(3*S*,5*S*,6*S*,8*R*,9*R*,10*S*)-3,6,9-trihydroxy-13(14)-labdean-16,15-olide-3-*O*-*β*-D-gluco-pyranoside (**96**)	Diterpenoid	No inhibitory activity on NO production in LPS-induced RAW 264.7 macrophages up to concentration of 100 μM.	*V*. *trifolia* L.	[82]
(3*S*,5*R*,10*S*)-3-[(*β*-D-glucopyranosyl)oxy]-labd-8,13-dien-16,15-olide (**97**) (3*S*,5*R*,10*S*)-3-hydroxy-labd-8,13-dien-16,15-olide (**98**)	Diterpenoids	Possessed inhibitory activities on LPS-induced NO production. Compounds **97** and **98** exhibited strong the activity of inhibition against NO production, and **98** was the strongest inhibitor with IC_50_ value of 15.8 ± 1.38 μM. Compound **98** also showed significant inhibition of IL-1β and IL-6 level. The anti-inflammatory mechanism of compound **98** was associated with its inhibition on iNOS, COX-2 and NF-κB signal pathways.	*V. negundo* var*. heterophylla*	[101]
9-epivitexnegundin (**99**)	Diterpenoid	Evaluated for its antimicrobial activity but the activity was not mentioned. No significant activity in cytotoxicity assays (IC_50_ > 100 µM) was reported.	*V. negundo* L.	[102,103]
Abieta-11(12)-ene-9*α*,13*α*-endoperoxide and malonyl derivative (**100** & **100a**) Abieta-11(12)-ene-9*β*,13*β*-endoperoxide and malonyl derivatives (**101** & **101a**) 9*αH*-manoyl oxide (**102**)	Diterpenoid	All compounds exhibited antimalarial activity compounds **101** and **101a** have the most activity with IC_50_ value of 1.2 µM ± 0.5 µM among others. Compound **101** was the most active with 54 folds more potent antimalarial than compound **100** due to the presence of *β*-peroxy bridge.	*V. rotundifolia*	[104]
Pinnatasterone (**103**)	Ecdysteroid	Exhibited weak activity against *M. domestica* larvae.	*V. pinnata* L.	[108]
Canescensterone (**104**) 24-epiabutasterone (**105**) (24*R*)-11*α*,20,24-trihydroxyecdysone (**106**) 11*α*,20,26-trihydroxyecdysone (**107**)	Ecdysteroid	-	*V. canescens* Kurz	[109,110,111]
26-hydroxypinnatasterone (**108**)	Ecdysteroid	-	*V. cymosa* Bertero ex Spreng.	[112]
24-epi-pinnatasterone (**109**) Scabrasterone (**110**)	Ecdysteroids	Exhibited very weak moulting activity in *Musca* bioassay.	*Vitex scabra* Wall. Ex Schauer	[113]
21-hydroxyshidasterone (**111**) 11*β*-hydroxy-20-deoxyshidasterone (**112**) 2,3-acetonide-24-hydroxyecdysone (**113**)	Ecdysteroids	Exhibited anti-inflammatory activity in rat paw oedema development assay at 100 mg/kg dose.	*V. doniana* Sweet	[114]
20,24-dihydroxy-24-hydroxymethylecdysone (**114**)	Ecdysteroid	This compund could be used in chemical defence against non-adapted herbivores. It also could be used as new and additional characteristic compound parameter in compound identification to reduce dereplication and false positives.	*V. cienkowskii*	[115]
Luteolin 6-C-(4″-methyl-6″-*O*-*trans*-caffeoylglucoside) (**115**) luteolin 6-C-(6″-*O*-*trans*-caffeoylglucoside) (**116**) luteolin 6-C-(2″-*O*-*trans*-caffeoylglucoside) (**117**) luteolin 7-*O*-(6″-*p*-benzoylglucoside) (**118**)	Flavonoids	-	*V. agnus*-*castus* L.	[118]
Vitegnoside (**119**)	Flavonoids	Exhibited antifungal activity against *T. mentagrophytes* and *C. neoformans* with MIC value of 6.25 μg/mL.	*V*. *negundo* L.	[25]
Vitecetin (**120**)	Flavonoids	Exhibited better antileishmanial activity than sodium antimonygluconate (SAG) with IC_50_ values of 2.4 and 58.5 mM for promastigote, and 0.93 and 36.2 mM for amastigotes. The compound was less toxic than SAG towards THP-1 with CC_50_ values of 123.7 mM and 364.3 mM, respectively.	*V. peduncularis* var. *cannobifolia*	[119]
2-(5-methoxyphenyl)-3,4′,5,7,8-trihydroxychroman-4-one (**121**) 2-(5′-methoxyphenyl) 4′,5,7-trihydroxy-3-methoxychromen-4-one (**122**) 2-(4′-hydroxyphenyl)-5-hydroxy 3,7- dimethoxy chromen-4-one (**123**) 2-(4-hydroxyphenyl)-3,5,7-trihydroxy chromen-4-one (**124**) 2-(3′,4′-dimethoxyphenyl)-7-hydroxychromen-4-one (**125**)	Flavonoid	All compounds exhibited moderate trypanocidal activity against *T. b rhodesiense*	*V. simplifocia*	[23]
4′-acetoxy-5-hydroxy-6,7-dimethoxyflavone (**126**)	Flavonoid	-	*V. penduncularis*	[120]
4,5-diethyl-3′-ethoxy-pyro flavone (**127**)	Flavonoid	Exhibited significant antifilarial activity in dose dependent manner.	*V*. *negundo* L.	[24]
Vitexdoin F (**128**)	Lignan	Exhibited stronger activity than ascorbic acid using DPPH radical-scavenging assays.	*V*. *negundo* L.	[123]
Vitexnegheteroin E (**129**)	Lignan	Exhibited antioxidant and inhibitory activities on lipopolysaccharide-induced NO.	*V. negundo* var. *heterophylla*	[124]
Vitexnegheteroin F (**130**)	Lignan	Exhibited moderate cytotoxic activities against human liver carcinoma (HepG2) cell lines.	*V. negundo* var. *heterophylla*	[124]
Vitexnegheteroin G (**131**)	Lignan	Exhibited antioxidant activities using ABTS scavenging activities.	*V. negundo* var. *heterophylla*	[124]
9-hydroxysesamin (**132**)	Lignan	-	*V. negundo* var. *heterophylla*	[124]
(3*R*,4*S*)-6-hydroxy4-(4-hydroxy- 3-methoxyphenyl)-5,7-dimethoxy-3,4-dihydro-2-naphthaldehyde-3a-*O*-*β*-d-glucopyranoside (**133**)	Lignan	-	*V. negundo* var. *heterophylla*	[125]
6,7,4′-trihydroxy-3′-methoxy-2,3-cycloligna-1,4-dien-2a,3a-olide (**134**)	Lignan	-	*V. negundo* var. *heterophylla*	[125]
6-hydroxy-4-(4-hydroxy-3-methoxyphenyl)-3-acetoxymethyl-7-methoxy-3,4-dihydro-2-naphthaldehyde (**135**)	Lignan	-	*V. negundo* var. *heterophylla*	[126]
3*α*-*O*-acetylvitedoin A (**136**)	Lignan	-	*V. negundo* var. *cannabifolia*	[126]
Vitekwangin A (**137**) Vitekwangin B (**138**)	Lignan	Both compounds showed minor cytotoxicity on RAW 264.7 cells with IC_50_ greater than 80 µM. At lower concentrations, compounds **137** and **138** exhibited minor effects of inhibitory on NO production.	*V. kwangsiensis* C.Pei	[127]
(9*R*)-*O*-*β*-D-glucopyranosyloxy-2,5-megastigmen-4-one (**139**) (3*S*,4*R*)-dihydroxy-7,8-dihydro-*β*-ionone 4-*O*-*β*-D-glucopyranoside (**140** & **140a**)	Monoterpenoids	All compounds showed anti-inflammatory activity and obvious inhibitory activity (IC_50_ > 100 μM), respectively.	*V. negundo* var*. heterophylla*	[101]
Cannabifolins **A**–**F** (**141**–**146**)	Triterpenoids	Compound **143** moderately inhibited NO production with IC_50_ value of 34.0 μM. Compounds **141**, **142**, **144** and **146** were inactive (<50% inhibition at 80 μM, the highest concentration tested). Compound **145** exhibited cytotoxicity on RAW 264.7 macrophages (cell viability <70% at 40 μM).	*V. negundo* var. *cannabifolia*	[130]
3*β*-hydroxy-30-al-urs-12-en-28-oic acid (**147**)	Triterpenoid	Exhibited cytotoxic activity against HL-60, SGC-7901, PANC-01, and Eca-109 cell lines.	*V. trifolia* var. *simplifocia*	[28]
Vitexnegheteroin H (**148**)	Triterpenoid	No inhibition on NO production.	*V. negundo* var. *heterophylla*	[124]
1*α*,*β*-dihydroxybauer-7-en-28-oic acid (**149**)	Triterpenoid	-	*V. doniana* Sweet	[131]
2*β*,3*β*,19*α*, 24-tetrahydroxy-23-norus-12-en- 28-oic acid (**150**)	Triterpenoid	-	*V. doniana* Sweet	[131]
Vitexnegheteroin A-D (**151**–**154**)	Phenolic glycosides	All compounds exhibited antioxidant and NO inhibitory activities.	*V. negundo* var. *heterophylla*	[132]
Methyl(6-*O*-4-hydroxybenzoyl)-*α*-D-Glucopyranoside (**155**)	Phenolic glycoside	Exhibited antioxidant and NO inhibitory activities.	*V. negundo* var. *heterophylla*	[132]
Breynioside A (**156**) 1,6-di-*O*-4-hydroxybenzoyl-*β*-D-glucopyranoside (**157**) Dunnianoside D (**158**)	Phenolic glycosides	Exhibited antioxidant and NO inhibitory activities.	*V. negundo* var. *heterophylla*	[132]
Vitexnegheteroin M (**159**)	Phenolic glycoside	Exhibited weak inhibitory effects on NO production with LPS-stimulate (IC_50_ > 100 µM) and no effect of cytotoxicity on BV-2 cells.	*V. negundo* var. *heterophylla*	[133]
Methyl 3-(2-(5-hydroxy-6-methoxy-4-oxo-4*H*-chromen-2-yl)ethylbenzoate (**160**) 3-(1-hydroxy-2-(5-hydroxy-6-methoxy-4-oxo-4*H*-chromen-2-yl)ethylbenzoic acid (**161**)	Chromone derivatives	The derivatives from *V*. *negundo* L. were able to reduce inflammation, and nociception, and could be used as a potential source of antinociceptive and anti-inflammatory candidates.	*V*. *negundo* L.	[135]

**Table 3:** Information on *Vitex* products available in the market.

Product Name, Unit Size, and Source (Website)	Country	Indication(s) for Use, Dosage Information, and Calculated Maximum Daily Intake (On Product Label)
Nature’s Answer *V. agnus*-*castus* L. 40 mg × 90 Capsules (https://www.naturesanswer.com) Accessed 1 June 2022	United States	This product may help to support woman’s hormonal balance. Suggested to be use as a supplement for diet. Take one capsule once a day with food or water.
Thompson’s-One-A-Day Vitex 1500 mg 60 Capsules (https://thompsonsherbals.com/en-au/products) Accessed 1 June 2022	New Zealand	This product may help relieve PMS and regulate menstrual cycle. For adults, take 1 capsule daily, first thing in the morning or as prescribed by a healthcare professional. Store below 30 °C in a dry place.
Solaray Vitex (Chastetree) 400 mg × 100 Vegetable Capsules (https://solaray.com) Accessed 1 June 2022	United States	*Vitex*, also typically known as chaste tree or chasteberry, is a purple-colored shrub of verbena family. It is native in Mediterranean region and have been historically used among women for over 2500 years. It should only be taken as directed. Take 1 capsule twice daily with a meal or glass of water.
Blackmores *V. agnus-castus* L. 660 mg × 40 tablets (https://www.blackmores.com.au) Accessed 1 June 2022	Australia	This product may help to regulate menstrual cycle, relieves breast pain and swelling. It is used traditionally in Western herbal medicine to help regulate the menstruation cycle. Only can be taken by adults. Take 1 tablet 3 times a day, or as prescribed by professionals. It should be taken before food.
Kordel’s Vitex 80 mg × 60 Capsules (https://www.kordels.co) Accessed 1 June 2022	Malaysia	Kordel’s vitex is traditional used to relieves premenstrual discomforts and to regulate menstruation. Only can be consume by adults. Take 1–2 capsules daily first thing in the morning, preferably before breakfast, or as directed by your health care professional.

**Table 4:** List of patents for *Vitex* species.

No.	Patent No./ Country	Title	Details	References
1	ES2190383A1 Spain	*Vitex agnus*-*castus* L. extract	The present invention provides a *Vitex agnus*-*castus* L. extract wherein the extract is obtained by extracting dried and pulverized fruits of the plant *Vitex* agnus castus with a 90–100% ethanol solvent, separating the extraction solution from the rest of the plant material, removing the solvent from the extraction solution, and recovering the extract. The present invention also provides for a dietary supplement comprising a *V. agnus*-*castus* L. extract having a linoleic acid content of at least ten weight percent by the composition and a calcium source and the use of the extract and dietary supplement to treat conditions particularly affecting women.	[153]
2	US8637099B2 United States.	Use of *Vitex agnus*-*castus* L. extracts for preparing a medicament	The present invention relates to the use of fruit extract from *Vitex agnus*-*castus* L. and/or one or more isolated and/or synthetically prepared bicyclic diterpenes from *V. agnus*-*castus* L. for the preparation of a medicament for the treatment of movement disorders.	[154]
3	USPP25914P3 United States.	*Vitex* plant named ‘PIIVAC-I’	A new and distinct cultivar of *Vitex* plant named ‘PIIVAC-I’, characterized by its compact, rounded to upright-spreading growth habit, dark green foliage, dark bluish-purple flowers, and resistance to leaf spot.	[155]
4	20160135348 United States.	*Vitex agnus-castus* plant named ‘V0509A-7’	The present innovation relates to a distinct and new cultivar of *Vitex agnus*-*castus* L. referred to as ‘V0509A-7’.	[156]

## Data Availability

Data available upon request.
